# Further Detail Concerning the Deep Learning Model for Mortality After Total Gastrectomy

**DOI:** 10.1002/ags3.70155

**Published:** 2025-12-21

**Authors:** Kentaro Goto, Haruku Fujita, Ryohei Onishi, Nobuaki Hoshino, Koya Hida

**Affiliations:** ^1^ Department of Surgery Kyoto University Kyoto Japan

Dear Editor,

1

We read with great interest the article by Fukuyo et al. titled “Deep Learning Model for Predicting Operative Mortality After Total Gastrectomy: Analysis of the Japanese National Clinical Database (NCD)” [1]. The application of deep learning to nationwide surgical registries is intriguing and represents an important direction in surgical data science. However, there are several methodological and statistical aspects that we found difficult to fully understand, and we would greatly appreciate clarification from the authors. In particular, we seek further information regarding the selection and application of evaluation metrics in model development.

First, a receiver operating characteristic (ROC) curve was employed to develop a prediction model. As surgeons, we may not be fully familiar with the detailed rationale for selecting the specific evaluation metrics in machine learning. ROC curves are widely used; however, in datasets with very low event rates, such as 1.2%, might the precision–recall curve provide a more informative perspective [2]? We would be grateful if the authors explained why the ROC curve was selected as the primary evaluation metric.

Second, the labels for the *x*‐ and *y*‐axes are not provided in figure 4. Could the authors kindly clarify what these axes represent? A calibration curve is generally understood to indicate better predictive performance when it aligns with the 45‐degree line; this figure appears to show a different pattern. We are interested in the authors' interpretation of the calibration results. Given that this study focused on rare events, we examined the right‐hand side of the calibration plot and noted that the observed mortality remained approximately 5%, even when the predicted probability was close to 80%. We would be grateful if the authors could confirm whether this interpretation is correct.

Third, although the discriminative ability (c‐index) of this model is lower than that of the previously reported NCD‐based model [3], could the authors kindly explain what advantages this model offers in terms of methodological innovation or potential clinical applicability?

Fourth, this study employed a relatively simple neural network architecture with four dense layers. The selection of an appropriate architecture for tabular clinical data can be challenging, and various approaches have been explored in recent studies. For example, boosting methods, such as XGBoost, LightGBM, and CatBoost, have been reported to perform favorably for structured data [4]. It would be helpful if the authors could elaborate on their rationale for selecting this architecture and indicate whether alternative approaches, such as boosting methods, were considered during model development.

Further detailed information regarding these aspects would provide a more comprehensive understanding of the study. We sincerely appreciate the opportunity to contribute to this discussion.

## Author Contributions

**Kentaro Goto:** conceptualization, investigation, writing – original draft. **Haruku Fujita:** conceptualization, investigation, writing – original draft. **Ryohei Onishi:** conceptualization, investigation, writing – original draft. **Nobuaki Hoshino:** supervision, writing – review and editing. **Koya Hida:** supervision, writing – review and editing. All authors have read and agreed to the published version of the manuscript.

## Funding

This work was supported by Japan Society for the Promotion of Science (JSPS) KAKENHI Grant‐in‐Aid for JSPS Fellows (grant no. 25KJ1576).

## Conflicts of Interest

The authors declare no conflicts of interest.

## Linked Articles

Deep Learning Model for Predicting Operative Mortality After Total Gastrectomy: Analysis of the Japanese National Clinical Database (NCD), https://doi.org/10.1002/ags3.70067.
